# Research Progress Towards and Prospects of Carbon Dots Derived from Tea and Chinese Medicinal Materials

**DOI:** 10.3390/nano15030171

**Published:** 2025-01-23

**Authors:** Xiaoxue Tang, Zhao Gong, Yan Lang, Hongyue Chen, Siqi Huang, Yuguang Lv

**Affiliations:** 1 College of Pharmacy, Jiamusi University, Jiamusi 154007, China; 2 Department of Rehabilitation, Wuyi University, Wuyishan 354301, China

**Keywords:** tea carbon dots, Chinese herbal medicine carbon dots, multiple applications, natural substances, special luminescence

## Abstract

This review focuses on the research progress related to carbon dots (CDs) derived from Chinese herbal medicines and tea, covering preparation methods, physicochemical properties, and application fields. It elaborates on preparation approaches like hydrothermal, solvothermal, microwave-assisted, and ultrasonic-assisted methods, and their influence on CDs’ structure and properties. It also explores CDs’ structural and optical properties. The application fields include antibacterial, sensing, bioimaging, photocatalysis, hemostasis, and energy. Carbon dots show antibacterial activity by destroying bacterial cell membranes, they can detect various substances in sensing, are important for bioimaging, degrade organic pollutants in photocatalysis, have hemostatic and anti-inflammatory effects, and can be used as battery anode materials. Despite progress, challenges remain in improving yield, quantum yield, property control, and understanding their mechanism of action. This review provides a reference for related research and looks ahead to future directions.

## 1. Introduction

**Figure 1:**
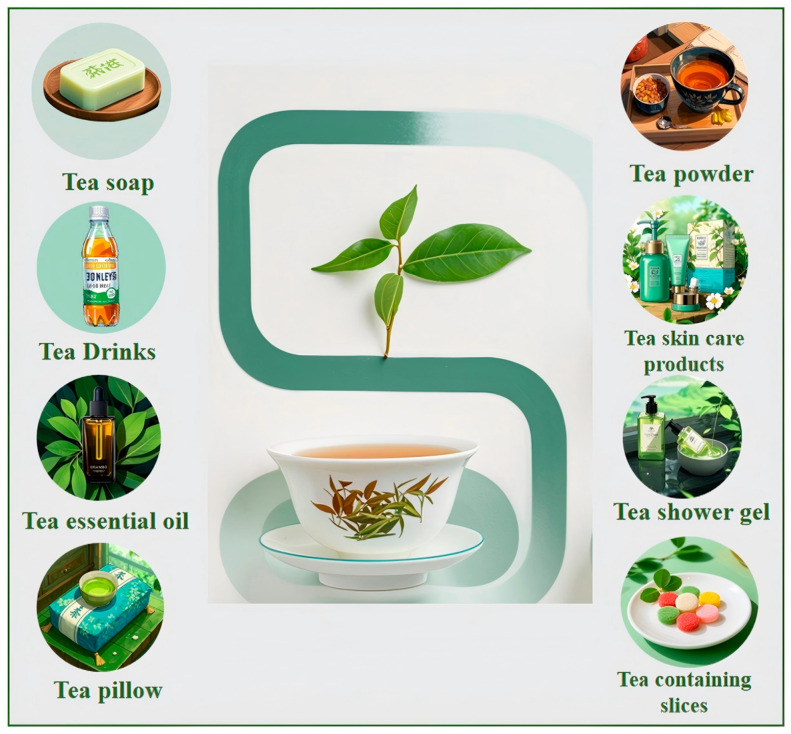
Tea leaves and their derived products.

In the vast expanse of nanomaterials science, CDs, a burgeoning class of carbon nanomaterials, have captured substantial attention ever since their discovery [1,2,3,4,5,6,7,8,9,10,11,12,13,14,15]. Their unique physicochemical properties, including remarkable optical performance, excellent biocompatibility, low toxicity, and a plenitude of surface functional groups, confer them vast application potential across a multitude of fields [16,17,18,19,20,21,22,23,24,25,26,27,28,29,30]. As nanotechnology steadily progresses, novel nanomaterials keep emerging [31,32,33,34,35,36,37,38,39]. CDs have thereby emerged as a focal point of research, covering areas from biomedicine to environmental science, and from the energy domain to sensor technology [40,41,42,43,44,45]. Both traditional Chinese medicines and tea, being natural products, are replete with organic constituents. Their pharmacological effects, which stem from the active substances within them, present distinctive carbon sources for fabricating novel carbon dots. In contrast to semiconductor quantum dots [46,47,48,49,50], CDs exhibit a higher resistance to photobleaching, a superior biocompatibility, and a lower toxicity [51,52,53,54,55,56,57,58,59,60,61,62,63,64,65,66]. Transforming waste tea residues and discarded Chinese herbal medicine into CDs augments the added value of waste biomass, in line with the principles of environmental conservation [67,68,69,70,71,72,73,74,75,76,77,78,79,80,81]. Moreover, the raw materials of tea- or traditional Chinese medicine-sourced CDs are mainly tea leaves and traditional Chinese herbal medicines, which possess specific medicinal values and chemical components. Tea leaves are rich in special components such as tea polyphenols and caffeine, providing a unique performance basis for tea—sourced CDs. Many CDs prepared from chemical raw materials may contain components that are harmful to organisms. For example, for some CDs synthesized from organic small molecules like benzene-based compounds, there may be unreacted toxic chemicals remaining on their surfaces. These substances may cause immune responses or cytotoxicity in organisms. As such, they cannot be applied to applications such as cell imaging and antibacterial use, which restricts the development direction of CDs. Comparing CDs sourced from traditional Chinese herbal medicines and tea leaves, the latter have a certain degree of biocompatibility due to the raw materials themselves, thus possessing potential advantages in biomedical applications. There are also differences in their fluorescence properties. The fluorescence characteristics of CDs not sourced from traditional Chinese medicines and tea leaves depend on synthetic raw materials and methods. Different raw materials and synthesis conditions can lead to changes in aspects such as fluorescence wavelength, intensity, and stability. Due to the special chemical components in their raw materials, tea- or traditional Chinese medicine-sourced CDs are endowed with unique fluorescence properties. The active ingredients in some traditional Chinese herbal medicines may interact with the surfaces of CDs, affecting their fluorescence emission. Traditional Chinese herbal medicines have anti-inflammatory, antibacterial, and other effects. The CDs prepared from them may exhibit better effects in applications such as bioimaging and treatment, have better biocompatibility, and overcome the drawback of the relatively strong cytotoxicity of chemically derived CDs. Their chemical stabilities also vary. The chemical stability of CDs not sourced from traditional Chinese medicines and tea leaves depends on their chemical structures, while tea- or traditional Chinese medicine-sourced CDs, due to possessing components with antioxidant, complexing, and other functions in their raw materials, have better chemical stability in certain environments (Figure 1).

**Figure 2:**
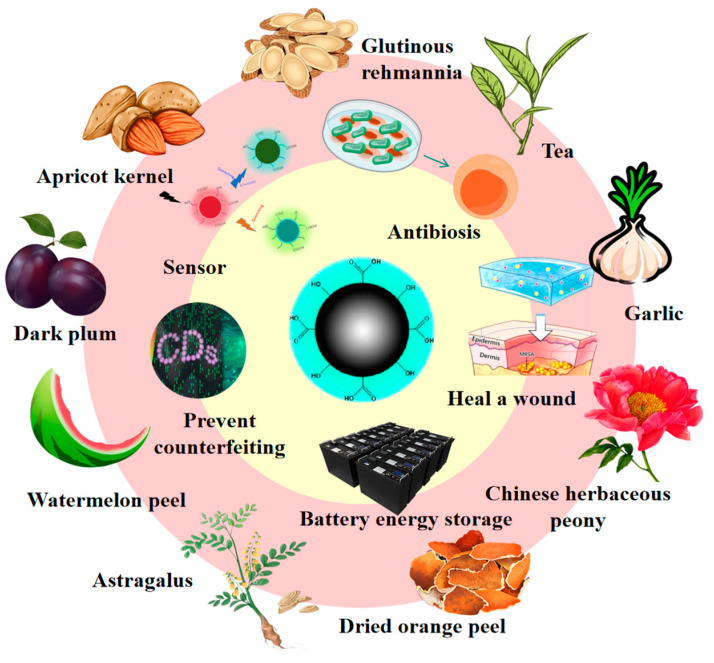
Carbon dots come from a variety of biomass sources, which can be used in different industries, including battery storage, sensors, photovoltaic devices, antioxidants, and anti-counterfeiting.

As a beverage that is widely consumed around the world, tea not only occupies an important position in culture and daily life, but also contains abundant components such as tea polyphenols, caffeine, and amino acids [82,83,84,85,86,87,88,89]. Using tea as a raw material to prepare carbon dots can not only facilitate the reuse of tea waste, but also produces carbon dots with potentially excellent properties. For example, tea-derived carbon dots can detect a variety of metal ions and biomolecules. Their antibacterial activity can be used to inhibit the growth and reproduction of bacteria, and they also perform well in biological imaging [83,84,85]. Traditional Chinese medicine has a long history and occupies an important position in traditional Chinese medicine. Its components are complex and diverse, including a variety of bioactive substances. Preparing carbon dots from traditional Chinese medicine can not only make full use of its natural ingredients, but may also endow the carbon dots with some special biological activities. For instance, some hemostatic Chinese herbal medicines are used to prepare carbon dots. Research has found that these carbon dots show hemostatic effects by influencing the endogenous and exogenous coagulation pathways. They also have anti-inflammatory effects and good biocompatibility. Biomass-based carbon dots have shown application value in multiple fields such as antibacterial, sensing, and biological imaging [90,91,92,93,94,95,96,97,98,99,100]. These provide new ideas for further research into new carbon dot materials (Figure 2).

From the perspective of preparation methods, there are diverse routes for the synthesis of biomass carbon dots [89,90,91,92,93,94,95,96,97,98,99,100,101,102,103,104,105,106]. The hydrothermal method, as a commonly used approach, can effectively produce carbon dots with good properties by promoting the carbonization and polymerization of organic substances in tea under a high temperature in a high-pressure water environment. This method, to some extent, simulates the geological processes in the natural environment and provides suitable conditions for the formation of carbon dots. The solvothermal method conducts similar reactions in an organic solvent system. By selecting different organic solvents, the properties of carbon dots can be finely tuned. The microwave-assisted method and the ultrasonic-assisted method utilize the special effects of microwave radiation and ultrasonic cavitation, respectively, achieving rapid and efficient carbon dot synthesis. Each of these methods has its own advantages and disadvantages, providing multiple options for the preparation of tea-derived carbon dots and also making it possible to further optimize their properties [107,108,109,110]. In terms of physicochemical properties, tea-derived carbon dots exhibit unique structural and optical characteristics [63,64]. Their structures are usually spherical or quasi-spherical, with a relatively narrow particle size distribution. These optical properties endow tea-derived carbon dots with potential application value in fields such as sensing and biological imaging [59]. The application fields of tea-derived carbon dots are quite extensive. In the sensing field, they can detect various metal ions, biomolecules, and environmental pollutants with a high sensitivity and high selectivity. Patra et al. [111] used mature green tea as the main carbon source to make CDs with good water solubility, photostability, and anti-interference from light emissions. These CDs can precisely detect Cr(VI) as the surface functional groups specifically interact with target analytes, causing fluorescence signal changes for quantitative analysis. In biological imaging, biocompatible and fluorescent tea-derived CDs are ideal biomarkers for cell and tissue imaging. Li et al. [104] prepared blue fluorescent CDs from mulberry leaves for intracellular bio-imaging and drug delivery, a powerful tool in biomedical research. In photocatalysis, Schomäcker et al. [64] employed tea leaves as templates to control composite material properties, providing more active sites for adsorption and efficient degradation. Tea-derived CDs also show antibacterial activity. Hu et al. [65] synthesized CDs from green tea leaves, which have strong antioxidant, antibacterial, and anti-inflammatory properties. They inhibit bacteria by disrupting cell membranes. Xie et al. [58] used waste tea residues to make carbon anodes with good energy storage in lithium/sodium-ion batteries. Carbon dots from high-temperature-carbonized Chinese herbal medicines are effective in treating diseases. For example, those from Coptis chinensis protect against snake-venom-induced acute kidney injury. Han et al. [103] found that jujube-derived carbon dots can promote red blood cell growth, potentially treating anemia and inspiring hemostatic drug development.

**Figure 3:**
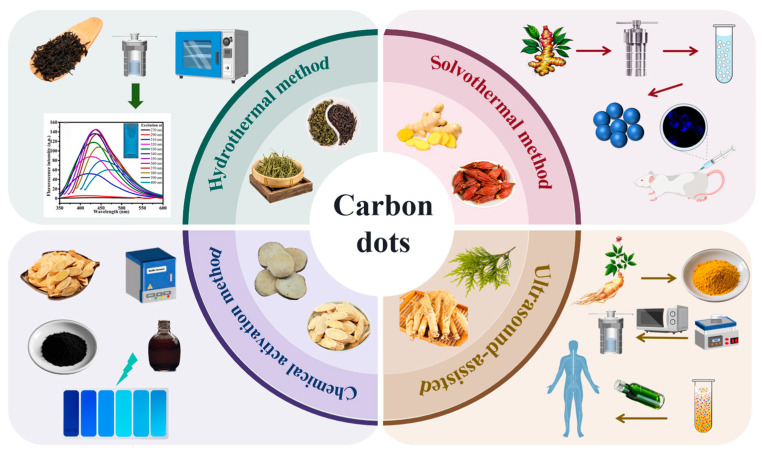
Carbon points and synthesis methods of different biomass sources (One of the illustrations is cited from Patra et al., 2024 [111]).

However, despite notable progress, challenges remain. In terms of preparation, improvements in the yield, fluorescence quantum yield, size control, and property regulation are needed. In the case of applications, a deeper understanding of interaction mechanisms and enhanced stability in real environments is required. This review comprehensively sums up the current research on the preparation, properties, and applications of tea-derived and Chinese herbal medicine-derived carbon dots, analyzes challenges, and looks ahead. By delving into these aspects, it aims to provide useful references for further research and application, driving the field forward (Figure 3).

## 2. Synthesis of Carbon Dots

### 2.1. Hydrothermal Method

Hydrothermal synthesis is one of the most common ways to prepare CDs from tea or Chinese herbal medicines. Here, tea leaves or waste are mixed with water, and then heated in a sealed autoclave under a specific temperature and pressure for a set time [104,105]. These hydrothermal conditions trigger the decomposition and carbonization of tea components, forming CDs. Patra et al. [111] took mature green tea leaves, washed, dried, and ground them into a powder, mixed with the powder with water, and reacted it at 200 °C for 10 h. After centrifugation and filtration, carbon dots with an average size of 5.2 ± 0.5 nm, good water solubility, photostability, and fluorescence properties were obtained. Khan et al. [59] used 1 g of green tea leaf powder, dispersed it in 50 mL of distilled water, and hydrothermally reacted the mixture at 180 °C for 8 h. The resulting spherical carbon dots had an average diameter of about 4.2 ± 0.825 nm after filtration and centrifugation. Chen et al. [63] soaked 1.5 g of waste tea in 30 mL of deionized water, boiled the mixture for 30 min at 80 °C, mixed the extract with 10 μL of ethylenediamine, and reacted it in a 150 °C Teflon-lined autoclave for 6 h. After cooling, centrifuging, and filtering, the solution was freeze-dried to obtain carbon dots. Djoko et al. [64] mixed urea and dried green tea leaf solutions, heated the mixture overnight at 80 °C in a water bath, dried, and calcined it at 500 °C for 2 h. Through further grinding, dispersing, ultrasonication, and centrifugation, carbon nanomaterial composites were acquired. These composites had an increased surface area, enhanced light absorption, an altered bandgap energy, and an improved carrier separation efficiency. Duarah et al. [62] processed factory tea waste, mixed it with water at 1:30 (solid–liquid ratio), and thermally reacted the mixture at 180–220 °C for 0–240 min. After cooling, filtering, and centrifuging, the filtrate was processed to obtain carbon dots. Compared to other plant-based carbon dot synthesis methods, the process used for green tea carbon dots is simpler. Their performance leans more towards antioxidant and anti-inflammatory functions, guiding the preparation of carbon dots with specific capabilities.

**Figure 4:**
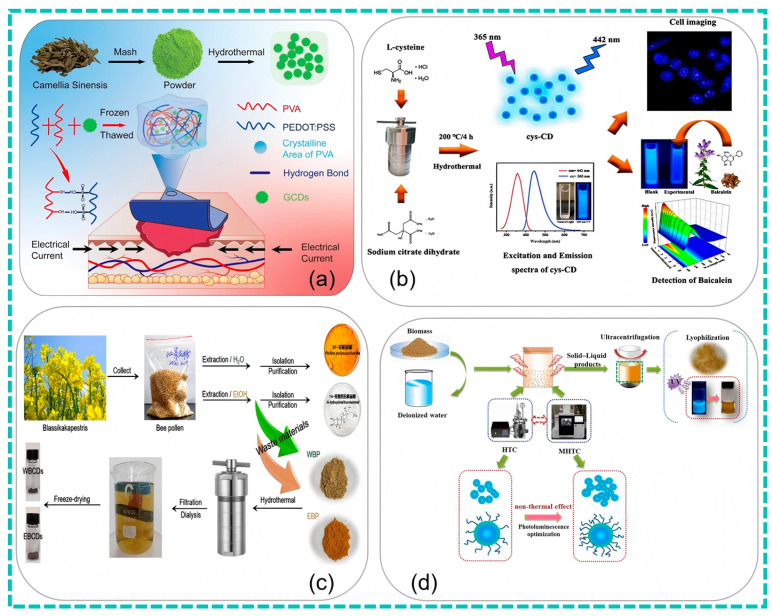
Preparation of conductive hydrogels for accelerating wound healing (**a**) (Hu et al., 2024 [99]); detection of baicalein CDs by hydrothermal synthesis (**b**) (Cheng et al., 2024 [2]); CDs from bee pollen using one-step hydrothermal method (**c**) (Shan et al., 2022 [81]); schematic production of hydrothermal CDs from soybean meal (**d**) (Guo et al., 2021 [67]).

In addition, for the preparation of CDs using other herbs as raw materials, Zhang et al. [100] crushed the dried herb residues, extracted them with ethanol, added boric acid, and then carried out a hydrothermal reaction at 200 °C for 8 h. After that, the product was concentrated, dialyzed, and freeze-dried to obtain boron-doped carbon dots. These carbon dots have an average diameter of approximately 2.8 nm, with abundant functional groups such as hydroxyl groups on the surface. They show a high selectivity and sensitivity towards Fe^3+^ (the detection limit is 1.08 μM), and can also be used for anti-counterfeiting and photodegradation. Lin et al. [105] synthesized fluorescent carbon dots with antibacterial activity using onions, ginger, garlic, fish, etc., as raw materials. The reaction was carried out at 200 °C for 8 h to obtain the carbon dots. Among them, the onion-derived carbon dots have relatively good antibacterial activity against Pseudomonas aeruginosa and a certain effect on the preservation of Atlantic mackerel. They can reduce drip loss and extend the shelf life of the mackerel by 2 days. Different from the previous synthesis of antibacterial carbon dots mainly using chemical reagents, this research uses food materials as precursors, expanding the source of raw materials for carbon dot synthesis. Compared with the traditional method of using chemical precursors to improve the quantum yield, this method using food materials is more biocompatible and safer (Figure 4).

### 2.2. Solvothermal Method

**Figure 5:**
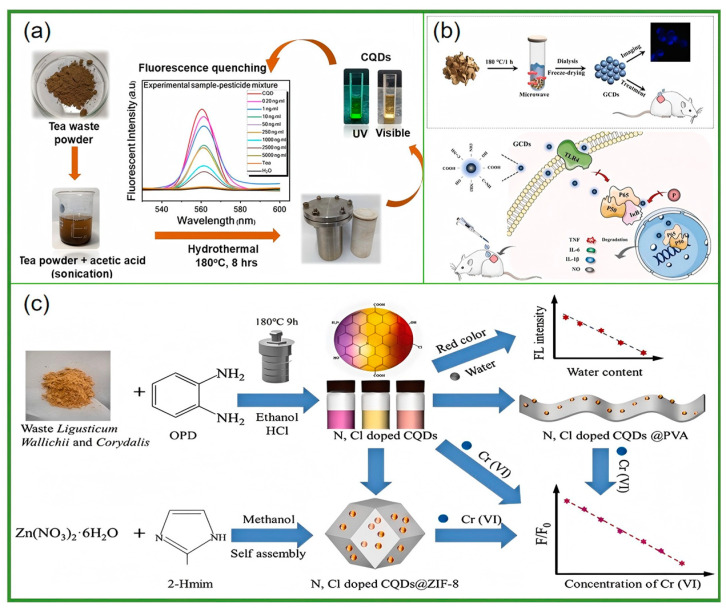
Waste tea CDs were prepared by solvent thermal method for pesticide testing (**a**) (Sinha et al., 2024 [60]); ginger-derived CDs accelerated wound healing (**b**) (Li et al., 2023 [104]); and one-pot solvent thermal preparation of seaweed CDs for detecting Cr (VI) (**c**) (Zhang et al., 2023 [100]).

The solvothermal method is an important approach to preparing carbon dots. In terms of the reaction system, the solvothermal method usually dissolves the carbon source in a specific solvent to form a uniform reaction system. Commonly used solvents include water, ethanol, and ethylene glycol. These solvents have good solubility and thermal stability, which can provide a suitable environment for the reaction. The carbon dots prepared using this method possess good crystallinity and dispersibility, enabling them to exhibit a better performance in subsequent applications. Ding et al. [66] used tea and o-phenylenediamine as raw materials and ethanol as the solvent. First, 0.8 g of white tea powder was refluxed in ethanol at 85 °C for 24 h. After cooling and filtration, 0.15 g of o-phenylenediamine was added, and a solvothermal reaction was carried out at 180 °C for 10 h. Then, nitrogen-doped carbon dots were obtained through column chromatography, rotary evaporation, and freeze-drying. These carbon dots have multiple emission peaks and can separately detect Hg^2+^ and H_2_O in two independent fluorescence channels, with a detection limit for Hg^2+^ as low as 1.20 nM. Sinha et al. [60] mixed 0.2 g of waste tea powder with 60 mL of 20% acetic acid solution, sonicated the mixture for 30 min, and then transferred it to a Teflon reactor. After reacting at 180 °C for 8 h, the mixture was cooled, centrifuged, and filtered twice to obtain carbon dots. The quantum yield of these carbon dots reached 40.05%, and they can be used to detect pesticides in tea. The detection limits for quinalphos, thiamethoxam, and propargite are 0.2, 1, and 10 ng/mL, respectively, and they can detect multiple pesticide mixtures simultaneously (Figure 5).

### 2.3. Microwave-Assisted

**Figure 6:**
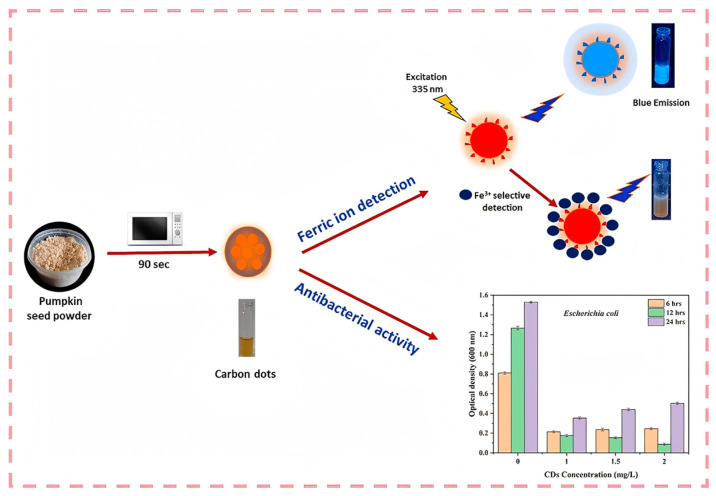
Schematic representation of the specific sensing of Fe and antimicrobials (III) from a microwave-assisted process in pumpkin seeds (Selvaraj et al., 2024 [110]).

Microwave-assisted synthesis is another effective approach for preparing tea-derived CDs. This method involves using microwave radiation to heat the reaction mixture. Compared with hydrothermal synthesis, it can significantly reduce the reaction time required. Li et al. [104] used ginger as the raw material. After slicing and grinding the ginger into powder, it was mixed with water and sonicated. Then, it was placed in a microwave synthesizer and reacted at 180 °C for 1 h. After filtration, dialysis, and freeze-drying operations, carbon dots were obtained. The study found that the average particle size of ginger carbon dots was 2.3 nm, and their surfaces were rich in functional groups such as -OH, -COOH, and -CONH_2_. They had good water solubility, dispersibility, and biocompatibility. These carbon dots could promote cell migration in vitro and accelerate wound healing in vivo. Guo et al. [112] adopted a microwave-assisted technique to produce CDs using eggshell membranes. The developed CDs had good water solubility with a quantum yield of 14%. Cheng et al. [113] used rose petals irradiated by microwaves as the carbon source, achieving a quantum yield of 13.45%. The obtained CDs could be used as a biosensor for tetracycline in human urine. Compared with the traditional hydrothermal method, the microwave-assisted hydrothermal method significantly shortens the preparation time, improves the synthesis efficiency, and does not require the use of organic reagents or surface-passivating agents, making it more environmentally friendly. Meanwhile, the carbon dots prepared using this method show promising application prospects in wound healing, providing a new approach towards the application of plant-derived carbon dots in the biomedical field (Figure 6).

### 2.4. Pyrolysis Method

**Figure 7:**
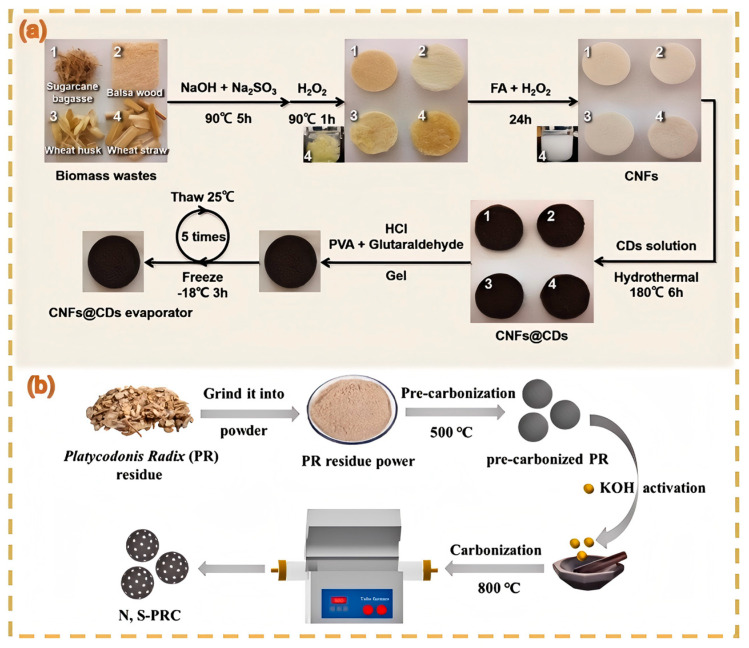
Astragalus CDs used for cell imaging (**a**) (Hao et al., 2022 [101]); pyrolysis of medicinal residues (hawthorn, chrysanthemum, peony cortex, hawthorn, and hair fruit) for battery energy storage (**b**) (Zou et al., 2024 [107]).

The pyrolysis method is a reliable and simple way to prepare green carbon dots. By heating carbon precursors and then separating and purifying them, carbon dots are obtained. Carbonization, an eco-friendly process, entails pyrolyzing organic materials in inert gas. Han et al. [103] carbonized traditional Chinese medicines like cattail pollen, lotus node, Cirsium japonicum charcoal, and hair at 300–450 °C. The resultant carbon dots had good biocompatibility and hemostatic effects, influencing both intrinsic and extrinsic coagulation pathways to shorten the clotting time. Chen et al. [114] processed astragalus membranaceus (washed, dried, and pulverized) via pyrolysis at 200 °C for 18 h. The obtained carbon dots were nearly spherical, with an average diameter of 10.14 ± 0.30 nm, a graphene-like structure, and −OH, −COOH groups on the surface. They had a 15.8% fluorescence quantum yield, plus good photostability, ionic strength stability, and thermal stability. They performed well in degrading malachite green (89% efficiency without light) and were used in HeLa cell imaging. Chai et al. [111] synthesized carbon dots from marigold and doped them with ethylenediamine, achieving a 7.84% quantum yield. Chen et al. [114] made CDs from carbonized watermelon rind, which were thermally stable at pH 2–11, 2 nm in diameter, and had a 7.1% quantum yield. Chen et al. [115] produced green CDs with a 19.3% quantum yield using carbonized sweet peppers. Bayda et al. [116] used black tea as a carbon precursor for CDs as drug delivery carriers. This method exploits the natural components and medicinal properties of Chinese herbs, offering a new source of biomass for carbon dot synthesis. The synthesized carbon dots have potential in biomedicine, with better biocompatibility and unique physiological activities than traditional chemical synthesis (Figure 7).

### 2.5. Generation of Fluorescent By-Products and Their Dialysis Treatment

Bottom-up synthesis methods such as the hydrothermal method, solvothermal method, and microwave-assisted synthesis usually produce some fluorescent by-products. These by-products mainly originate from incompletely carbonized precursors or partially carbonized intermediate products. The presence of fluorescent by-products can affect the fluorescence properties and application effects of CDs, so effective removal methods are required. Dialysis is a commonly used method to remove fluorescent by-products. Through dialysis, macromolecular CDs can be separated from small-molecule by-products in the solution, improving the purity and fluorescence stability of CDs. Dialysis is usually carried out using a dialysis membrane with an appropriate molecular weight cut-off (MWCO), and the dialysis time is usually 24–48 h to ensure the complete removal of by-products. CDs prepared from different precursors may require different dialysis conditions. For example, CDs from traditional Chinese medicines/teas may need a longer dialysis to remove more fluorescent by-products. Chen et al. [117] soaked 1.5 g of waste tea in 30 mL of deionized water. After a series of reactions, the resulting solution was cooled, centrifuged, and filtered. Subsequently, impurities and possible fluorescent by-products were removed by dialysis, and finally, carbon dots were obtained by freeze-drying. When Han et al. [103] prepared CDs by carbonizing traditional Chinese medicinal materials such as Typha angustifolia and Nelumbinis Nodus Rhizomatis, the dialysis step was not mentioned. This may be because the pyrolysis process was relatively simple with fewer by-products, or other unmentioned purification methods were used. It could be that fewer fluorescent by-products were generated under their synthesis conditions, and centrifugation and filtration were sufficient to meet the requirements for the purity of CDs in the study, or perhaps the research focus was on the application performance of CDs, with insufficient assessment of the impact of by-products. Although dialysis is an effective method for removing fluorescent by-products, there are still some challenges, such as long dialysis times and high costs. Future research should explore more efficient removal methods, such as nanofiltration and ultrafiltration technologies, to improve the purity and fluorescence performance of CDs.

## 3. Properties of Carbon Dots Derived from Tea and Chinese Herbal Medicines

### 3.1. Optical Properties

**Figure 8:**
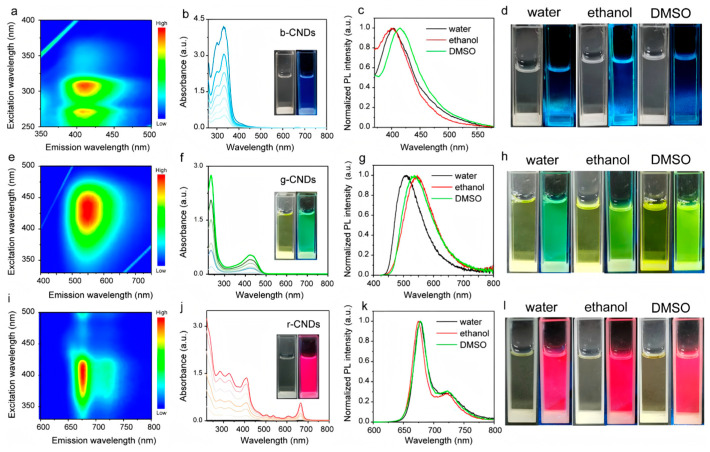
Honeysuckle, turmeric, and Perilla leaves synthesized using CDs based on the blue, green, and red fluorescence of biomass, as shown in the Figure, (Zhao et al., 2021 [90]). ((**a**) Excitation-emission contour plots of the b-CNDs; (**b**) UV-vis absorption spectra of the b-CNDs, The insets are the images of CNDs under sunlight and 365 nm excitation; (**c**) The PL spectra of modified b-CNDs are dispersed in water, ethanol and DMSO; (**d**) the corresponding images under sunlight and 365 nm excitation; (**e**) Excitation-emission contour plots of the g-CNDs; (**f**) UV-vis absorption spectra of the g-CNDs; (**g**) PL spectra of g-CNDs dispersed in different solvents; (**h**) Image of g-CNDs dispersed in different solvents; (**i**) Excitation-emission contour plots of the r-CNDs; (**j**) UV-vis absorption spectra of the r-CNDs; (**k**) PL spectra of r-CNDs dispersed in different solvents; (**l**) Image of r-CNDs dispersed in different solvents).

Biomass-derived CDs exhibit unique advantages and potential in the optical field. Generally, they can display extremely intense fluorescence emissions in the visible light region, a characteristic that gives them an important position in numerous optical applications. Biomass-derived CDs have broad application prospects in fields such as biological imaging, photocatalysis, and sensors. It is worth noting that the fluorescence emission wavelengths and intensities of CDs are not fixed but can be precisely regulated by skillfully adjusting the synthesis conditions. When the reaction temperature rises, it may accelerate the carbonization process of the biomass raw materials, causing changes in the structure of the carbon dots and thus affecting their fluorescence emission properties. A longer reaction time may give the carbon dots more time to grow and mature, making their structures more complete, which has a positive impact on their fluorescence emission wavelengths and intensities. Different biomass raw materials and other added precursor substances will introduce different elements and functional groups during the synthesis process, and these differences will be directly reflected in the final properties of the CDs. For example, Ding et al. [66] developed multifunctional label-free nitrogen-doped ratiometric fluorescence CDs from tea extracts and o-phenylenediamine, which demonstrated two completely independent ratiometric fluorescence channels for the detection of Hg^2+^ and H_2_O. This achievement provides a new and efficient detection method for environmental monitoring and analysis (Figure 8 and Table 1).

### 3.2. Structural Properties

**Figure 9:**
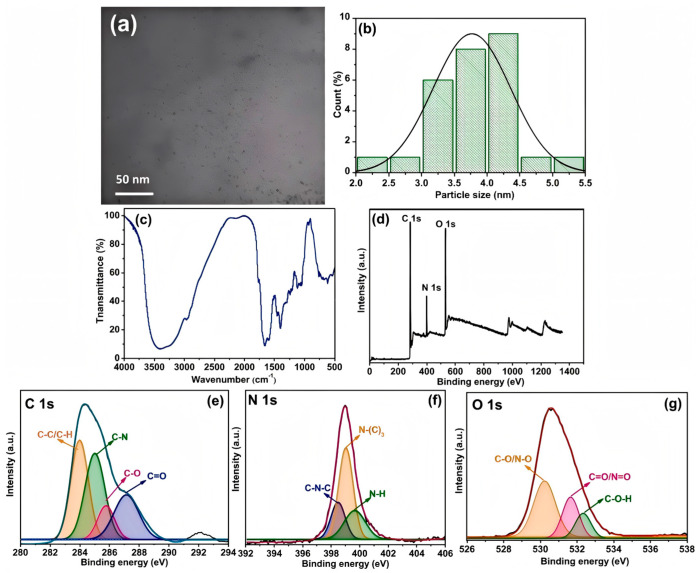
Schematic representation of the structural characterization of CDs prepared from green tea powder. (**a**) TEM image of the CDs and (**b**) size histogram of the CDs with a curve fitted to the data using a Gaussian model, (**c**) FTIR spectrum, (**d**) XPS full survey scan, and corresponding high-resolution scans of (**e**) C 1 s, (**f**) N 1 s, and (**g**) O 1 s. (Khan et al., 2023 [59]).

In the in-depth exploration of biomass-derived CDs, researchers have employed a variety of advanced and sophisticated technical means, including transmission electron microscopy (TEM), X-ray diffraction (XRD), and Raman spectroscopy, aiming to comprehensively and thoroughly analyze their structural properties. When observing biomass-derived CDs with TEM, the obtained images clearly show their unique morphological features. These CDs usually present with a spherical or quasi-spherical appearance and have remarkable narrow size distribution characteristics [96,97,98,99]. Their particle morphology is very close to the ideal spherical shape, with a high size consistency. Under high-resolution TEM imaging, almost no obvious size deviations or irregular shapes can be seen. The average diameter is usually in the range of 2–10 nm. This morphological structure leads to a more stable and uniform performance. This size range gives the CDs unique advantages in many application scenarios. In the biomedical field, such a tiny size enables them to easily penetrate biological membranes and enter the interior of cells, thus achieving effective monitoring of and interventions in intracellular biological processes. Meanwhile, CDs of this size can serve as ideal nanofillers, which are uniformly dispersed in the matrix material and effectively improve the properties of the matrix material. When analyzing biomass-derived CDs using XRD technology, their XRD patterns usually show broad diffraction peaks, which are a significant indication of their amorphous nature. Raman spectroscopy also plays a crucial role in research on this topic. It has successfully revealed the presence of sp^2^- and sp^3^-hybridized carbon atoms in these CDs. From the perspective of chemical bonds, the functional mechanisms of sp^2^- and sp^3^-hybridized carbon atoms in the structure of CDs can be explained in detail. It has been pointed out that sp^2^-hybridized carbon atoms usually participate in forming a conjugated system, endowing CDs with certain optical and electrical properties, while sp^3^-hybridized carbon atoms play an important role in maintaining the structural stability of CDs. Adjusting the ratio of sp^2^- and sp^3^-hybridized carbon atoms can effectively regulate the properties of CDs, such as their fluorescence emission wavelength, intensity, and electrical conductivity, providing new ideas and methods for optimizing the performance of CDs (Figure 9).

### 3.3. Surface Properties

CDs derived from tea and Chinese herbal medicines exhibit unique performance advantages in the field of materials science, which are largely attributed to the rich and diverse functional groups on their surfaces [118,121,124,125,126,127,128,129]. Among them, functional groups such as hydroxyl, carboxyl, and amine groups are particularly prominent. The presence of these functional groups endows CDs with a series of excellent properties. As a common functional group, the hydroxyl group has strong hydrophilicity. It can form hydrogen bonds with water molecules, thus conferring a good water solubility to CDs. For example, specific molecules or groups can be attached to the surface of carbon dots, enabling them to specifically recognize and bind to target cells. By connecting hydrophilic groups, the dispersibility of CDs in an aqueous environment can be enhanced, making it easier for them to approach the cell surface and increasing the opportunity for contact with cells. Meanwhile, the introduction of targeting groups, such as antibody fragments and aptamers, enables CDs to specifically recognize and bind to specific receptors on the cell surface, thereby achieving precise imaging of specific cell types. This property enables CDs to be uniformly dispersed in an aqueous solution system, providing convenient conditions for their applications in fields such as biomedicine and environmental detection. The carboxyl functional group further enhances the water solubility of CDs and can also participate in chemical reactions, interacting with other molecules or ions. In sensing applications, the carboxyl group can specifically bind to specific analytes, causing changes in the optical or electrical properties of CDs and achieving a highly sensitive detection of the analytes. The amine group can interact with biomolecules in living organisms, reducing the immune response to and toxic effects of CDs on the organism. In drug delivery systems, the amine group can be linked or modified with drug molecules to achieve targeted transport and the controlled release of drugs. The presence of these functional groups also enables tea-derived CDs to interact with various analytes, greatly expanding their potential in sensing applications.

In addition, optimizing the synthesis conditions for carbon dots derived from tea and traditional Chinese medicine can regulate their physicochemical properties, thereby improving their application effects in bioimaging. For example, parameters such as the ratio of carbon source to nitrogen source, reaction time, and temperature can be adjusted to obtain carbon dots with better fluorescence properties and biocompatibility. At the same time, new synthesis methods such as the microwave method and hydrothermal method can also be explored to improve the efficiency and quality of prepared carbon dots. Adjusting the synthesis conditions is also an important way to enhance the interaction between CDs and cells. By changing synthesis parameters such as reaction temperature, time, type and concentration of precursors, the size, surface charge, and surface functional group distribution of CDs can be regulated. Smaller-sized CDs often have a better cell penetration ability. By precisely controlling the size of CDs through optimizing the synthesis conditions, they can enter cells more efficiently. Meanwhile, adjusting the surface charge so that the surface of CDs carries a charge complementary to that of the cell surface can enhance the electrostatic attraction between the two, promoting the binding and uptake of CDs by cells, thus improving the imaging effect and opening up broader application prospects for bioimaging research.

## 4. Applications of Carbon Dots

### 4.1. Sensing Applications

**Figure 10:**
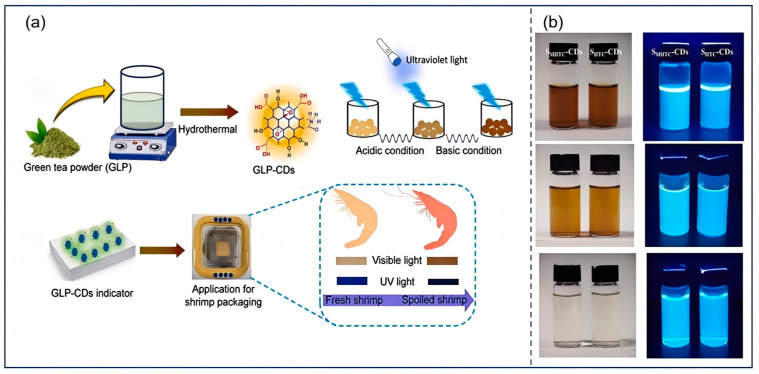
CDs were prepared from green tea powder for real-time freshness detection of shrimp packaging (**a**) (Khan et al., 2023 [59]); CDs with blue fluorescence (**b**) (Guo et al., 2021 [67]).

Thanks to their unique optical and surface traits, tea-derived CDs are promising for detecting various analytes. Their fluorescence can be modulated upon interacting with target molecules, enabling sensitive and selective detection. These CDs are widely used to detect metal ions. Jin et al. [121] designed novel orange-red fluorescent N-CDs from peony seed residue and o-phenylenediamine via a 180 °C, 8 h hydrothermal process. The N-CDs had good water solubility, excellent fluorescence, and a high photostability. Their 594 nm fluorescence intensity varied linearly with Fe^3+^ from 0.0 to 50.0 μM, with a 20 nM detection limit. For quick Fe^3+^ sensing, two solid-phase test strips and nanofiber membranes compounded with N-CDs were made. Xie et al. [124] synthesized fully water-soluble nitrogen-doped N-CDs from highland barley (carbon source) and ethylenediamine (nitrogen source) using a green hydrothermal method. TEM and XRD showed their graphite amorphous structure and narrow size distribution. XPS and FT-IR verified the presence of lattice doping and surface hydrophilic groups. The N-CDs emitted intense 480 nm blue fluorescence, with a 14.4% quantum yield, and could sensitively detect Hg^2+^ (10–160 μM, 0.48 μM limit). He et al. [118] synthesized spherical biomass nitrogen-doped blue fluorescent CDs (1–6 nm) from longan peel via one-step hydrothermal treatment for rapid Cr^6+^ detection in food and packaging. The carboxyl, hydroxyl, and amino groups on the surfaces of the CDs ensured good water dispersibility. A fluorescence sensor based on CDs’ fluorescence was built, showing a linear relationship between fluorescence quenching rate and Cr^6+^ concentration. Ding et al. [125] proposed an eco-friendly method to prepare CDs from pig ribs and feces via acid carbonization and hydrothermal treatment. These CDs had great fluorescence and biocompatibility. A sensing platform based on their coordination fluorescence could sensitively and selectively detect dimethoate (0.064 μM limit, 0.15–5.0 μM linear range). It was fast, convenient, and could directly detect dimethoate in water, with 93–105% recovery and 2–6% RSD. Wang et al. [126] made biomass CDs from orange peel, ginkgo, tung, and magnolia leaves via a simple hydrothermal method. These CDs had a uniform size, great water solubility and stability, and similar optical and surface features. They were sensitive to Fe^3+^ (0.2–100 μM), with a 0.073 μM detection limit. The detection mechanism often involves complex formation between metal ions and surface functional groups, which can quench or enhance fluorescence (Figure 10).

### 4.2. Biological Imaging Applications

**Figure 11:**
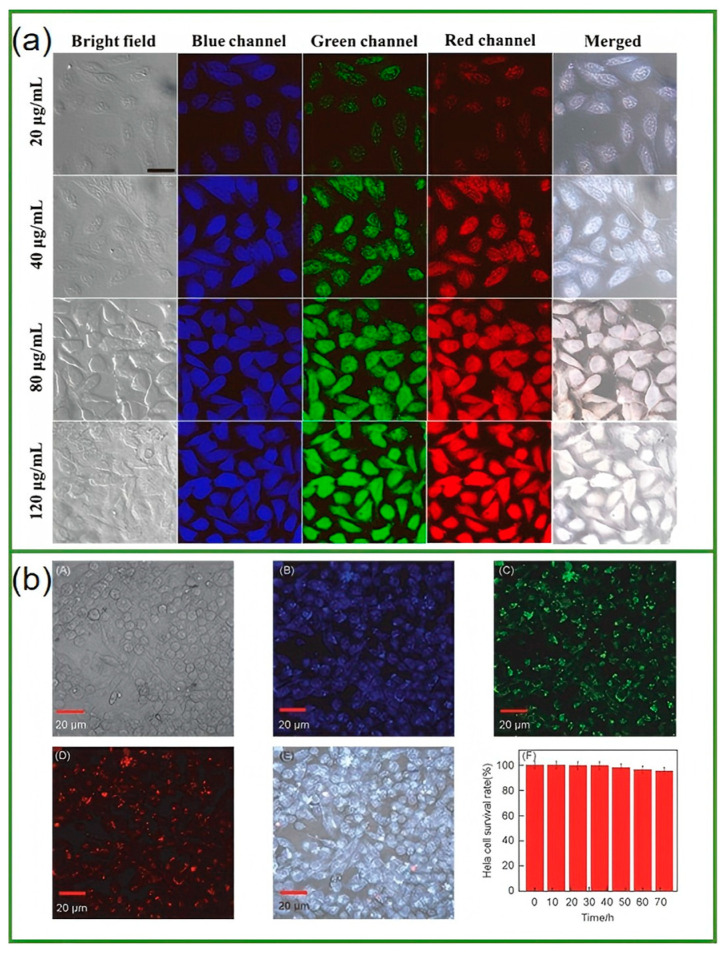
CLSM fluorescence images of HeLa cells incubated with different concentrations of N, P-CDs (20, 40, 80, and 120 μg mL⁻^1^). The scale bar is 20 µm. (**a**) Taken from Qin et al., 2023 [108]; fluorescence microscopic images of HeLa cells incubated with CDs at 37 °C. (**b**) (Hao et al., 2022 [101]). ((**A**) Bright field image; (**B**–**D**) fluorescence images taken on exciter filters: excitation 340 nm(blue), excitation 460 nm (green), excitation 520 nm (red), respectively; (**E**) merged images of HeLa cells in bright field and fluorescence mode; (**F**) HeLa cell survival rate).

The excellent fluorescence properties and biocompatibility of biomass tea-derived CDs make them highly suitable for biological imaging applications, allowing for the visualization of biological structures and processes at the cellular and tissue levels. The derived CDs can be internalized by cells, and their fluorescence can be used to label and visualize cells. The small size of CDs enables them to enter cells easily without causing significant damage. The fluorescence emission of CDs can be used to distinguish different cell types. Qin et al. [108] used confocal laser scanning microscopy (CLSM) to observe the intracellular distribution of N, P-CDs from mango peel. The excitation wavelengths were set at 340 nm (blue channel), 460 nm (green channel), and 520 nm (red channel), respectively, and the corresponding emission light was collected. The results showed that N, P-CDs could penetrate all the incubated HeLa cells, and the fluorescence signal covered almost the entire cell area, clearly outlining the cell contour as shown in Figure 11a. This indicates that N, P-CDs can not only penetrate the cell membrane but also enter the cell nucleus. Quantitative analysis of the fluorescence intensity showed that the intracellular fluorescence intensity gradually increased with the increase in the concentration of CDs, demonstrating the excellent stability of CDs during the process of cell internalization. The images of HeLa cells and the fluorescence signals under different excitation channels could be well integrated, making them effective multi-color fluorescent markers in bioimaging. Hao et al. [101] used an Olympus BX51 microscope for fluorescence microscopy observation. The excitation wavelength was set at 320 nm, and the emission light was detected at 405 nm to display the confocal images. In the merged images, the combination of fluorescence signals from different channels provided a comprehensive view of the distribution of carbon dots within HeLa cells. The strong fluorescence intensity both inside and outside the cells further confirmed the high fluorescence efficiency of carbon dots. In addition, HeLa cells remained viable and maintained a normal morphology after incubation with CDs, indicating that CDs have a low toxicity towards cells, making them promising candidates for bioimaging applications.

**Figure 12:**
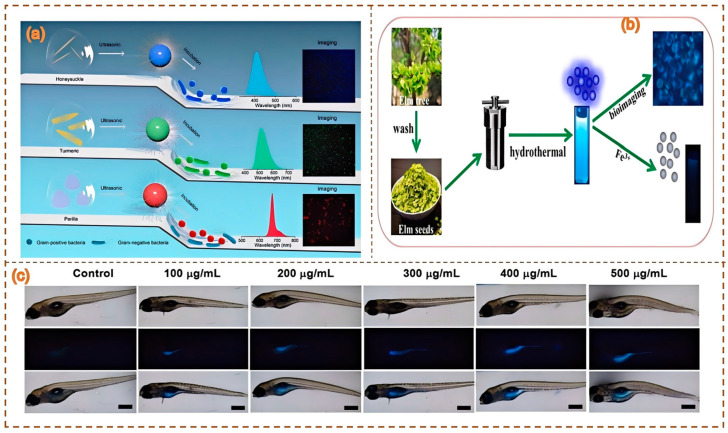
Lonicerae, turmeric, and Perilla leaves used to create synthetic CDs for cell imaging (**a**) (Zhao et al., 2021 [90]); schematics of fluorescent elm seed synthetic CDs and applications in Fe^3+^ detection and bioimaging (**b**) (Zhang et al., 2023 [100]); peach leaf CDs for in vitro cell culture and fluorescence imaging in zebrafish (**c**) (Ren et al., 2023 [130]).

Thota et al. [127] proposed a simple green method for synthesizing highly fluorescent carbon dots from peanut shells (GNS). The biocompatibility of GCD was confirmed by cell viability tests, and it was suitable for yeast cell imaging. Korram et al. [128] adopted a simple one-step carbonization method for the sustainable hydrothermal synthesis of fluorescent blue nitrogen-doped carbon quantum dots (NCQDs) using banana petioles obtained from biomass waste. A paper-based assay was developed to quantitatively estimate Fe^3+^, with a limit of detection (LOD) value of 0.47 nM for the solution assay and 0.94 nM for the paper-based assay. Using smartphone-based readings, the biological imaging study of banana leaf cells using NCQDs revealed the selective staining of stomatal openings on the leaves. Annisa et al. [129] nanostructured and functionalized insoluble phytochemicals such as chlorophyll and curcumin into CDs, indicating that Andrographis paniculata–urea-based CDs were the best particles for cell penetration, which was related to changes in the surface charge and functional groups of the CDs. The optimal dose was 12.5 μg/mL for three consecutive treatment hours for the biological imaging assay. Shahraki et al. [123] developed CDs using banana peels as carbon precursors via a simple hydrothermal method. The cytotoxicity properties and cell internalization of CDs in breast cancer cells (MCF-7) were further investigated. The results showed that cell viability decreased significantly with increasing dose, reaching 50% (IC_50_) at a concentration of 128 μg/mL. The rind of watermelon is called “Watermelon Green Tunic” in traditional Chinese medicine. After removing the soft inner part, washing it, and drying it in the sun, it can be used as a traditional Chinese medicine. Zhou et al. [52] pyrolyzed watermelon rind at a low temperature and then filtered it. The obtained CDs possess strong blue luminescent properties, excellent water solubility, and good stability in solutions with a wide range of pH values and high salinity. In the HeLa cell imaging experiment, these CDs successfully labeled the cells and emitted clear fluorescence signals (Figure 12).

### 4.3. Photocatalytic Applications

**Figure 13:**
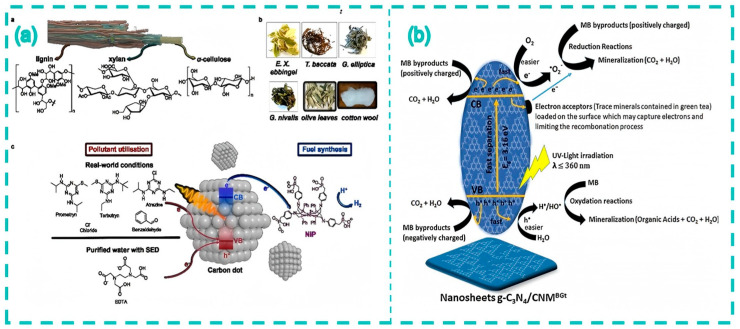
CDs using lignocellulose for the photocatalytic degradation of organic pollutants (**a**) (Achilleos et al., 2024 [131]); CDs from green tea for the photodegradation of pollutants (**b**) (Djoko et al., 2020 [64]).

The presence of functional groups on the surface of the derived CDs enables them to absorb light and generate reactive oxygen species (ROS), such as superoxide anions (O_2_^−^) and hydroxyl radicals (·OH). These ROS can react with organic pollutants, degrading them into smaller and less harmful molecules. For instance, in the presence of tea-derived CDs and under appropriate light irradiation, organic dyes like methylene blue have been effectively degraded. CDs act as photosensitizers, absorbing photons and transferring energy to generate ROS, which attack the dye molecules, break their chemical bonds, and lead to their decomposition. Sharma et al. [131] efficiently synthesized CDs from pear juice, which showed an effective degradation of methylene blue under visible light. These CDs exhibited excellent light absorption and electron transfer properties, contributing to an enhanced photocatalytic activity. Within just 130 min, the degradation rate of methylene blue reached 95%. Tyagi et al. [119] developed a CDs/TiO_2_ composite catalyst using CDs extracted from lemon peel waste. The developed CDs/TiO_2_ catalyst effectively degraded methylene blue dye. These findings underscore the crucial role of CDs in the photocatalytic field (Figure 13).

### 4.4. Antibacterial Applications

**Figure 14:**
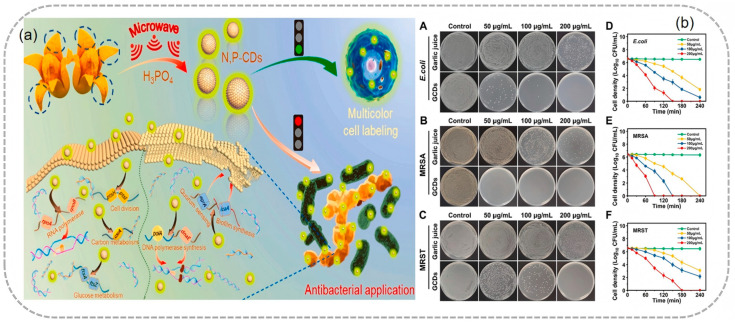
CDs prepared from mango peel were used to explore the antimicrobial mechanism of *E. coli* and *S. aureus* (**a**) (Hua et al., 2023 [108]), and hydrothermal synthetic CDs derived from garlic were used for antimicrobial activity (**b**) [(**A**–**F**) Bactericidal effect of garlic juice and GCDs on (**A**) *E. coli*, (**B**) MRSA, and (**C**) MRST. Time-kill curves of GCDs for (**D**) *E. coli*, (**E**) MRSA, and (**F**) MRST. (n = 3, mean ± SD)] (Selvaraj et al., 2024 [110]).

Mechanisms of Antibacterial Action: The antibacterial activity of tea-derived CDs is based on multiple mechanisms. A negative electric potential allows CDs to interact with the positively charged bacterial cell membranes via electrostatic attraction. Oxygen-rich functional groups, such as hydroxyl and carboxyl groups, can further strengthen this interaction. Once attached to the surface of bacterial cells, CDs can disrupt the integrity of the cell membranes, leading to the leakage of cell contents and ultimately cell death. In addition, CDs can also generate ROS when exposed to light or in the presence of certain substances, which can cause oxidative damage to bacterial cells. Pricilla et al. synthesized CDs with an average diameter of 6.3 nm from the medicinal seed extract of Syzygium jambos using a one-pot hydrothermal synthesis method. CDs at a concentration of 500 µg/mL showed antibacterial activity against four common pathogens. *Staphylococcus aureus* and *Staphylococcus epidermidis* were completely eradicated by the CDs within 12 h, while *Escherichia coli* and Klebsiella pneumoniae required 24 h. Hua et al. [108] developed negatively charged, tunable multicolor nitrogen–phosphorus co-doped CDs (N,P-CD) from mango peel. The negatively charged N,P-CD has inherent antibacterial activity against *Escherichia coli* and *Staphylococcus aureus*, and the antibacterial mechanism was studied by real-time quantitative polymerase chain reaction (RT-qPCR). This demonstrates the potential of biomass-derived CDs as broad-spectrum antibacterial agents, which can be used in applications such as food preservation, coatings for medical devices, and water treatment to control bacterial contamination (Figure 14).

### 4.5. Hemostasis

**Figure 15:**
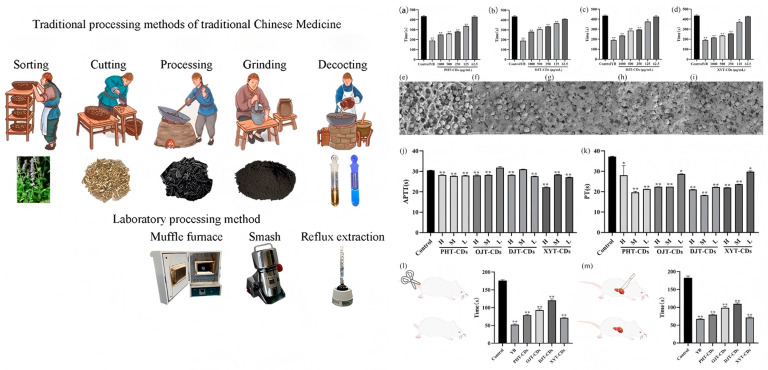
Schematic diagram of hemostatic effects by using traditional Chinese medicine as a biomass carbon source in the synthesis of CDs (Han et al., 2023 [103]). [subfigures (**a**–**m**): (**a**−**d**) Histogram of CDs in vitro hemostasis experiments, respectively, (**e**) SEM of the blood clot in the blank control, (**f**–**i**) SEM of the blood clot after CDs administration, respectively. (**j**) APTT histogram. (**k**) PT histogram. (**l**) Histogram of bleeding time in the rat tail cutting experiment. (**m**) Histogram of bleeding time in the rat liver laceration experiment. Compared with the control group, ** *p* < 0.01, * *p* < 0.05].

Han et al. [117] prepared carbon dots using cattail pollen, lotus node, Cirsium japonicum charcoal, and hair as raw materials by mimicking the carbonization process of traditional Chinese medicine hemostatic materials. It was found that these carbon dots could significantly shorten the clotting time by affecting the intrinsic and extrinsic coagulation pathways. In in vitro clotting experiments, they showed a high-concentration clotting ability comparable to that of Yunnan Baiyao. Moreover, they effectively reduced the bleeding time in rat tail-cut and liver laceration models, along with having good biocompatibility and anti-inflammatory activity. This provides a new perspective on the preparation of traditional Chinese medicine hemostatic carbon dots, indicating their potential value in the research and development of hemostatic materials. Mo et al. [132] carbonized ginger. Oxidizing ginger can strengthen its warming properties, reduce its irritation, and improve its dispersibility. It can be used in warming moxibustion devices, relieving diarrhea, warming the meridians, stopping bleeding, and treating uterine cold bleeding, hematochezia, and stomachache. It can also promote the dissolution and absorption of glycoside components, indirectly enhancing the hemostatic effect. However, the specific mechanism of action of carbon dots in the hemostasis of carbonized medicine still requires in-depth research. Zhang et al. [133] were the first to prove that water extracted from Schizonepeta tenuifolia (SHC) can be used to produce carbon dots via an improved pyrolysis method. Based on the evaluation of the coagulation parameters in mice, these effects may be related to the activation of the extrinsic coagulation activity and the fibrinogen (FIB) system. These studies prepared carbon dots using different raw materials and methods and explored their applications in hemostasis and wound healing [122], demonstrating the application potential of carbon dots in the biomedical field. Subsequent research is continuously deepening our understanding of their mechanisms of action and expanding the scope of their application (Figure 15).

### 4.6. Energy-Related Applications

**Figure 16:**
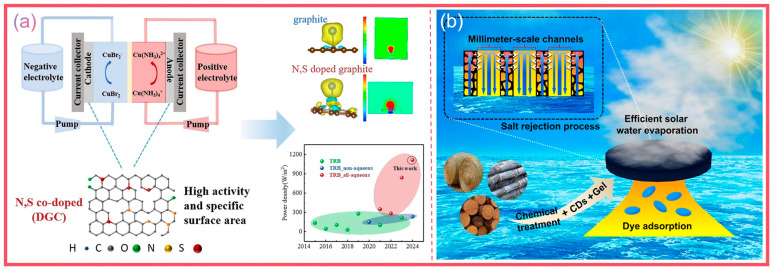
Co-doped ginger-derived porous carbon electrode (**a**) (Li et al., 2024 [134]); CDs prepared using bagasse, wheat husk, and wheat straw for battery energy storage (**b**) (Wang et al., 2022 [126]).

Li et al. [134] conducted research on the preparation of N, S site-activated porous carbon materials for symmetric supercapacitors using Platycodon grandiflorum root residues. After washing, drying, and pulverizing the residues of traditional Chinese medicines such as Platycodon grandiflorum roots, chrysanthemums, tree peony barks, Clinopodium chinense, and papayas, they were first carbonized at 500 °C for 1 h in a nitrogen environment to obtain N, S site-activated porous carbon materials (N, S-PRC). A symmetrical supercapacitor was assembled with N, S-PRC-4 and PVA/KOH gel electrolyte. Its working voltage window was 0–1.4 V. The CV curves maintained regular rectangles at different scanning rates, and the GCD curves were almost symmetrical at different current densities, indicating a fast charge transfer and good kinetic reversibility. This device had a relatively high specific capacitance at different current densities. After 10,000 charge–discharge cycles, the capacitance retention rate reached 90.2%, and the energy density reached 15.4 W h kg^−1^ at 250 W kg^−1^. Dong et al. [135] pyrolyzed CDs generated from durian peels. By introducing CDs as dopants, the electrode characteristics and supercapacitance performance were significantly improved. The specific capacitance of the composite electrode created using polyvinylidene fluoride was 60 F g^−1^ (Figure 16 and Table 2).

## 5. Discussion

### 5.1. Multifunctional Applications and Combinations

By combining different properties and applications, tea-derived CDs have the potential to be developed into multifunctional materials. For example, CDs can be designed to possess both sensing and photocatalytic capabilities, or to be used for simultaneous biological imaging and drug delivery. This requires a deeper understanding of how to integrate and optimize these diverse functions within a single material. Some functional groups or ligands can enable multiple interactions and activities. For instance, specific biomolecules for biological imaging and drug delivery can be attached while maintaining the sensing or photocatalytic properties of the CDs through other surface modifications. Combining tea-derived CDs with other materials can also enhance their performance and bring about new applications. Incorporating CDs into a polymer matrix can improve the mechanical properties of the resulting composite while retaining the unique optical and chemical properties of the CDs. This can be useful in applications such as flexible sensors or bioactive coatings. In photocatalysis, combining biomass-derived CDs with other semiconductors or catalysts can generate heterojunction structures, thus improving charge separation and overall photocatalytic efficiency.

### 5.2. Toxicity and Biocompatibility

Studies on biomass-derived CDs show good biocompatibility in vitro. However, comprehensive in vivo toxicity studies are needed to evaluate their long-term effects on living organisms, such as their accumulation in organs and their impacts on organ functions and immune responses. Long-term monitoring of biodistribution and toxicity is required during application. To improve the biocompatibility of CDs, it is necessary to understand the influencing factors and study their interactions with biomolecules and the impacts on cell membranes and intracellular processes. Optimizing the surface chemistry and size can enhance their compatibility and reduce adverse effects. Standardized testing methods and guidelines are also required to ensure safety and efficacy. In terms of application, research on the long-term safety of and metabolic pathways used by carbon dots in living organisms is insufficient, and regulatory standards for food applications remain to be perfected. These issues impede the rapid transformation of laboratory research into practical products.

### 5.3. Challenges and Future Directions

Although progress has been made in creating CDs derived from tea or Chinese herbal medicines, there are still challenges affecting their development and application. These include optimizing the synthesis process, understanding the sensing mechanisms, improving performance, expanding toxicity and biocompatibility studies, and establishing regulations. There are opportunities in the development of multifunctional CDs [119,123,130,131,134,135,136,156] and their combination with other materials. Scalability and cost-effectiveness are crucial, and environmental impacts and sustainability also need to be considered. At present, largescale and cost-effective production technologies are not yet mature, and ensuring their stability in complex real-world systems such as biological fluids or environmental matrices remains a major obstacle. Looking ahead, the prospects of these carbon dots are full of possibilities. In the energy field, they may bring about a revolution in battery electrodes and supercapacitors. In the catalytic field, they may play a key role in promoting sustainable reactions such as photocatalytic water splitting and carbon dioxide reduction. Regarding their applications in the food and cosmetics fields, adding CDs to food packaging materials can inhibit the growth of microorganisms and extend the shelf life of food. Alternatively, carbon dots can be made into preservatives for the preservation of foods such as fruits and vegetables, which is expected to improve product quality, safety, and efficacy. Some Chinese herbal medicines have the effects of whitening and freckle-removing, and carbon dots derived from them may also have similar functions. In the future, we can explore the application of these carbon dots in cosmetics to develop cosmetics with whitening and freckle-removing functions. In the emerging field of quantum technology, carbon dots may become key components in quantum sensing and communication. They can serve as photon sources or quantum entanglement sources in quantum communication for the transmission and processing of quantum information. In the future, research can be conducted on the application of carbon dots derived from tea and Chinese herbal medicines in quantum communication to improve the efficiency and security of quantum communication. In addition, their fluorescence properties can change due to external environmental factors, and they can be applied to the preparation of smart fluorescent switch materials for use in fields such as information storage, anti-counterfeiting labels, and environmental monitoring. Finally, by compounding CDs with materials such as polymers, smart materials with shape-memory functions can be prepared for applications in biomedical, aerospace, intelligent robotics, and other fields, which contributes to the development of novel functional materials and high-performance devices. Further research efforts should focus on overcoming existing challenges to unlock these potential applications.

Traditional methods for carbon dot synthesis are often plagued by low yields. The problem of low synthesis yields also exists in the synthesis of CDs from traditional Chinese medicines and teas. However, the yield can be significantly improved by selecting appropriate precursors and optimizing reaction conditions. At present, numerous studies have reported various methods for synthesizing CDs from traditional Chinese medicines or teas. Nevertheless, the synthesis yields vary greatly and are comprehensively affected by multiple factors. In terms of raw materials, the components of different teas or traditional Chinese medicinal materials vary significantly, and the content and types of their organic components affect the synthesis yield. The synthesis method is also crucial. For example, in the hydrothermal method, the combination of reaction temperature, time, and pressure affects the yield. To increase the synthesis yield, researchers are actively exploring strategies, including optimizing process parameters, developing or improving synthesis methods, and studying the relationship between raw materials and yield. Although there are problems with the synthesis yield, in-depth research and optimization are expected to solve these problems and promote the development of this field. Future research should focus on the analysis of the synthesis reaction mechanism. By using advanced characterization techniques and theoretical calculation methods, the rules influencing the effect of the raw material components and synthesis conditions on the structure and properties of carbon dots can be clarified to achieve customized synthesis. Strengthening the research on the biosafety and pharmacokinetics of carbon dots, conducting in vivo experiments to provide a clinical basis, and promoting the formulation of relevant regulations and standards will facilitate the application of CDs in the field of homologous substances of medicine and food. It is expected that this will lead to breakthroughs and development opportunities in aspects such as food safety protection and disease treatment, providing strong support for human health and upgrading the food industry.

### 5.4. Comparison with CDs Derived from Other Wastes and Biomass

#### 5.4.1. Uniqueness of Composition and Properties

Traditional Chinese medicines and tea are rich in a variety of special components, which is an important foundation for the unique properties of the CDs prepared from them. Components such as polyphenols and caffeine which are abundant in tea can participate in the formation of carbon dots during the synthesis of CDs, endowing CDs with good antioxidant properties. Research shows that tea-derived CDs have a high scavenging ability for superoxide radicals and hydroxyl radicals and perform excellently in antioxidant applications. The components of traditional Chinese medicines are more complex and diverse. Different Chinese herbal medicines contain various active ingredients such as alkaloids, flavonoids, and terpenoids. Taking astragalus membranaceus as an example, the CDs prepared from it not only have fluorescent properties but also retain some of the biological activities of astragalus membranaceus, such as immunomodulatory effects, which provides more possibilities for its application in the biomedical field. In contrast, the components of general waste materials and biomass are relatively simple, and the CDs prepared from them have relatively conventional properties.

#### 5.4.2. Advantages in Synthesis Process and Conditions

Traditional Chinese medicines and tea exhibit certain advantages in the synthesis of CDs. On the one hand, the complexity of their components means that there is no need to add excessive additional reagents during synthesis, reducing synthesis steps and costs, and lowering the risk of introducing impurities. On the other hand, the synthesis conditions are relatively mild. The hydrothermal method is one of the commonly used methods for preparing CDs. When using tea or traditional Chinese medicines for hydrothermal reactions, the required temperature and pressure conditions are usually lower than those required for some other biomasses. This is not only beneficial for energy conservation but also better preserves the active ingredients in the raw materials, thus affecting the properties of CDs. In contrast, some other waste materials and biomasses need to undergo processes such as recycling and impurity removal, which increases the difficulty and cost of synthesis.

#### 5.4.3. Targeted Advantages in Application Fields

CDs prepared from traditional Chinese medicines and tea possess clear targeted advantages for various application fields. In the biomedical field, due to their good biocompatibility and the potential biological activities they may carry, these CDs have broad application prospects in areas such as bioimaging, drug delivery, disease diagnosis, and treatment. Research has reported that CDs prepared from certain hemostatic traditional Chinese medicines can effectively shorten the clotting time in in vivo experiments and can be used to develop new hemostatic materials. In the environmental field, the abundant functional groups on the surface of tea-derived CDs endow them with a strong adsorption capacity for some heavy metal ions, enabling them to be used for environmental monitoring and pollutant removal. In contrast, CDs prepared from other waste materials and biomasses may require more modifications and adaptations to meet the requirements of the specific fields in which they are to be applied. In conclusion, CDs prepared from traditional Chinese medicines and tea have unique advantages over those prepared from other waste materials and biomasses in terms of their composition, synthesis process, and application fields.

## 6. Conclusions

CDs derived from tea or Chinese herbal medicines are a promising class of nanomaterials with multiple potential applications. Synthesizing CDs from tea leaves or tea waste is an environmentally friendly and sustainable method that makes full use of the organic components in tea. The complex interactions between biomass-derived CDs and analytes affect the CDs’ sensing sensitivity and selectivity. Particularly in metal ion sensing, the functional groups and coordination geometries of CDs are crucial to the binding affinity. High-selectivity detection faces challenges because CDs are easily interfered with by multiple analytes. Future research should focus on strategies to improve the selectivity of CDs, such as ligand functionalization, while developing real-time and in situ detection capabilities to address the deficiencies of existing detection methods. Although there has been some progress in tea-derived CDs, challenges still need to be overcome in terms of developing applications, including optimizing synthesis, understanding the relationships between properties and parameters, enhancing sensitivity and selectivity, improving light absorption performance, and expanding toxicity and biocompatibility studies. Continuous research and development will help unleash the potential of CDs and promote their applications.

## Figures and Tables

**Table 1:** Precursors for carbon dot preparation and their optical and application properties.

Precursors	FL Color	Applied Ex/Em (nm)	Fluorescence Quantum Yield (%)	Applications	Ref.
Tea	Blue	420/512	22.26 ± 1.10	Antibacterial activity	[117]
Tea	Green	310/499	-	Fluorescence detection	[63]
Tea	Blue	360/438	-	Fluorescence detection	[65]
Tea	-	450/530	-	Antibacterial activity	[98]
Tea	Green	360/440	11.70	Fluorescence detection	[118]
Tea	Blue	340/480	18.50	Photocatalysis	[119]
Tea	Green	280/561	40.05	Fluorescence detection	[60]
Tea	Green	380/415	5.66	Antibacterial activity	[99]
Tea	-	270/431	-	Photocatalysis	[120]
Tea	Blue	320/410	14.8	Fluorescence detection	[60]
Tea	Green	386.5/480	7.88	Antioxidation	[101]
Cole flowers	Blue	360/512	4.80	Cell imaging; Sensor; Plant growth agent	[83]
Ginger	Blue	340/430	-	Food preservation; Antibacterial activity	[105]
Ginger	Blue	360/430	6.00	Cell imaging; Antioxidation	[104]
Turkey fig	Red	405/673	10.68	Antioxidation	[108]
Dark seeds	Blue	340/673	-	Antioxidation	[109]
Soya bean	Blue	400/485	4.62	Fluorescence detection	[121]
Ligusticum wallichii and Corydalis	Purple; yellow; red	326/364; 380/544; 578/646	3.58	Fluorescence detection	[122]
Garlic	Blue	330/442	-	Antioxidation	[101]
Honeysuckle	Blue	330/410	11.00	Cell imaging	[90]
Carcuma longa	Green	430/520	12.00	Cell imaging	[90]
Folia perillae acutae	Red	420/670	28.00	Cell imaging	[90]
Pollen Typhae	-	352/442	-	Stop bleeding	[104]
Rhizoma alismatis	Blue	320/405	15.80	Cell imaging; Photocatalysis	[123]
Scutellariae Radix	-	409/503	3.26	Stop bleeding	[122]
Different herbs	Green	360/445	4.00	Cell imaging; Sensor	[100]

**Table 2:** The techniques used for the synthesis of CDs from various precursors relevant to different applications.

Precursors	Methods	Additive	Reaction Conditions	Size	Applications	Ref.
Ginger	Hydrothermal	Carbinol	200 °C for 8 h	1.95 nm	Antibacterial activity	[108]
Green tea	Hydrothermal	Water	150 °C for 4 h	0.21 nm	Wound Healing	[104]
Green tea	Solvothermal	o-diaminobenzene	85 °C for 24 h	0.20 nm	Fluorescence detection	[63]
Tea	Hydrothermal	Water	150 °C for 5 h	2.90 nm	Photocatalysis; Sensor	[64]
Indonesian’s Spent Tea Leaves	Hydrothermal carbonization	Water	200 °C for 40 h	0.388 nm	Anode Materials	[57]
Waste Tea	Hydrothermal	Ethylic acid	180 °C for 8 h	0.22 nm	Sensor	[60]
Green tea	Hydrothermal	Water	180 °C for 8 h	2–5.5 nm	Real-time monitoring of shrimp freshness	[59]
Green tea	Solvothermal	Carbinol	80 °C for 12 h	1–5 nm	Photocatalysis	[136]
Waste tea	Hydrothermal	Ethanediamine	150 °C for 6 h	2.2 nm	Antioxidation; Sensor	[65]
Mesua ferrea Linn leaf	Hydrothermal	Alcohol	150 °C for 5 h	3.20 nm	Photocatalysis	[101]
Green tea	Chemical activation	concentrated sulfuric acid	20 °C for 20 h	5.00 nm	Sensor	[66]
Mango skin	Hydrothermal	Water	200 °C for 20 h	3.83 nm	Antimicrobial; imaging	[108]
Barley	Solvothermal	Alcohol	200 °C for 8 h	0.22 nm	Cell imaging; Sensor	[121]
Sanguisorbae Radix	Hydrothermal	Water	100 °C for 1 h	3–10 nm	Hemostatic Effects	[111]
Green tea	Solvothermal	Choline chloride	200 °C for 8 h	7.64 nm	Fluorescence sensor	[137]
Ginkgo leaf	Hydrothermal	Water	180 °C for 8 h	2.625 nm	Photocatalysis	[113]
Lotus root	Pyrolysis	Water	350 °C for 1 h	2.89 nm	Cell imaging; Sensor	[138]
Rhizoma chuanxiong	Solvothermal	Alcohol	200 °C for 8 h	0.28 nm	Cell imaging; Sensor	[128]
Phellodendri cortex carbonisatus	Pyrolysis	Water	350 °C for 1 h	1.2–4.8 nm	Antihemorrhagic	[122]
Cirsium setosum carbonisata	Pyrolysis	Water	350 °C for 1 h	1.0–5.0 nm	Antihemorrhagic	[133]
Paeoniae radix alba	Pyrolysis	Water	350 °C for 1 h	1.0–2.4 nm	Antioxidation	[139]
Semen prunus	Hydrothermal	Water	200 °C for 8 h	0.33 nm	Cell imaging; Sensor	[138]
Peanut shell	Hydrothermal	Water	220 °C for 10 h	0.22 nm	Cell imaging; Sensor	[127]
Ginger-derived	Hydrothermal	Microwave	180 °C for 1 h	2.3 nm	Wound healing	[104]
Oil-tea camellia	Solvothermal	Alcohol	200 °C for 8 h	2–8 nm	Sensor	[138]
Radices rehmanniae	Hydrothermal	Water	180 °C for 10 h	2–10 nm	Cell imaging; Sensor	[129]
Maize straw	Hydrothermal	Water	270 °C for 10 min	0.18 nm	Biological imaging; Sensor	[140]
Lemon juice	Hydrothermal	Water	350 °C for 3 h	5.7 nm	Biological imaging	[141]
Sugarcane	Hydrothermal	Water	150 °C for 8 h	4.1 nm	Biological imaging	[142]
Almond	Solvothermal	Alcohol	40 °C for 12 h	2–10 nm	Biological imaging	[143]
Calotropis gigantea	Hydrothermal	Water	200 °C for 8 h	2.7–10.4 nm	Sensor	[144]
Wheat	Hydrothermal	Water	180 °C for 36 h	2–6 nm	Photocatalysis	[145]
Rubber	Hydrothermal	Water	200 °C for 6 h	4.4 nm	Photocatalysis	[146]
Oil brown empty fruit	Hydrothermal	Water	270 °C for 2 h	4.2 nm	Sensor	[147]
Carcuma longa	Hydrothermal	Water	180 °C for 24 h	2.85 nm	Sensor	[148]
Rice bran	Hydrothermal	Water	200 °C for 4 h	2.96 nm	Photocatalysis	[149]
Guava leaf	Hydrothermal	Water	160 °C for 1 h	4–7 nm	Photocatalysis	[150]
Rice husk	Hydrothermal	Water	190 °C for 4 h	4.8 nm	Photocatalysis	[151]
Hylocereus undatus	Hydrothermal	Water	180 °C for 12 h	2.5 nm	Photocatalysis	[152]
Lignin	Hydrothermal	Water	180 °C for 8 h	3.6 nm	Photocatalysis	[153]
Onion	Hydrothermal	Water	180 °C for 36 h	0.2 nm	Sensor	[154]
Cassava stalks	Hydrothermal	Water	180 °C for 36 h	1–5 nm	Sensor	[120]
Spinacia oleracea	Hydrothermal	Water	180 °C for 36 h	2–10 nm	Sensor; Biological imaging	[155]
